# Dyslexia and dysgraphia of primary progressive aphasia in Chinese: A systematic review

**DOI:** 10.3389/fneur.2022.1025660

**Published:** 2022-12-06

**Authors:** Junyan Liu, Shoko Ota, Nobuko Kawakami, Shigenori Kanno, Kyoko Suzuki

**Affiliations:** Department of Behavioral Neurology and Cognitive Neuroscience, Tohoku University Graduate School of Medicine, Sendai, Japan

**Keywords:** primary progressive aphasia, Chinese-speaking patients, reading errors, tonal errors, writing errors

## Abstract

**Introduction:**

Currently, little is known about Chinese-speaking primary progressive aphasia (PPA) patients compared to patients who speak Indo-European languages. We examined the demographics and clinical manifestations, particularly reading and writing characteristics, of Chinese patients with PPA over the last two decades to establish a comprehensive profile and improve diagnosis and care.

**Methods:**

We reviewed the demographic features, clinical manifestations, and radiological features of Chinese-speaking PPA patients from 56 articles published since 1994. We then summarized the specific reading and writing errors of Chinese-speaking patients.

**Results:**

The average age of onset for Chinese-speaking patients was in their early 60's, and there were slightly more male patients than female patients. The core symptoms and images of Chinese-speaking patients were similar to those of patients who speak Indo-European languages. Reading and writing error patterns differed due to Chinese's distinct tone and orthography. The types of reading errors reported in Chinese-speaking patients with PPA included tonal errors, regularization errors, visually related errors, semantic errors, phonological errors, unrelated errors, and non-response. Among these errors, regularization errors were the most common in semantic variant PPA, and tonal errors were specific to Chinese. Writing errors mainly consisted of non-character errors (stroke, radical/component, visual, pictograph, dyskinetic errors, and spatial errors), phonologically plausible errors, orthographically similar errors, semantic errors, compound word errors, sequence errors, unrelated errors, and non-response.

**Conclusion:**

This paper provides the latest comprehensive demographic information and unique presentations on the reading and writing of Chinese-speaking patients with PPA. More detailed studies are needed to address the frequency of errors in reading and writing and their anatomical substrates.

## Introduction

Primary progressive aphasia (PPA) is a clinical syndrome that mainly impairs language function and results from the selective neurodegeneration of the language network. Deficits in language are insidious and progress gradually, presenting as the most prominent clinical feature in the absence of marked impairments in other cognitive and behavioral domains at symptom onset and in the initial phases of the disease (1, 2). According to language phenotype, imaging, and pathology, PPA has been categorized into three types: (1) the non-fluent/agrammatic variant (nfvPPA), which is characterized by agrammatism in language production and effortful speech, and predominant left posterior fronto-insular atrophy/hypometabolism; (2) the semantic variant (svPPA), which is characterized by anomia and single-word comprehension deficits, and predominant anterior temporal lobe atrophy/hypometabolism; and (3) the logopenic variant (lvPPA), which has remarkable features of word retrieval and sentence repetition deficits, and predominant left posterior perisylvian or parietal atrophy/hypometabolism (2).

To date, most studies on PPA have focused on patients speaking Indo-European languages, while there is limited knowledge of the presentations of patients using Chinese, a logographical group of languages completely different from alphabetic languages. In addition, studies on patients using Chinese have been confined to case reports and retrospective studies with small sample sizes. Since PPA is a heterogeneous group of neurodegenerative diseases that selectively damage the language network in the brain, it is reasonable to question whether differences in ethnicities and languages have an impact on the prevalence and manifestations of PPA. To refine the clinical practice paradigm worldwide and pave the way for prompt diagnosis and comprehensive management, it is crucial to incorporate other languages into PPA research. Therefore, we aimed to summarize the demographic data, clinical manifestations, neuropsychological test results, and neuroradiological features of Chinese speakers with PPA from the 56 articles published since 1994 to describe the profile of PPA in Chinese speakers. A review article about the demographics of Chinese patients with frontotemporal dementia (FTD) published in 2012 included 14 patients with nfvPPA and svPPA (3). Here, we provide an up-to-date and comprehensive systematic review of the characteristics of all the three types of PPA in Chinese speakers. Furthermore, we observed that Chinese patients with PPA exhibit some specific reading and writing errors not observed in the Indo-European languages due to the presence of tone and logographic orthography in Chinese (4–11), which will be detailed here.

## Chinese tone and script

The syllable of standard Chinese pronunciation, also known as Chinese Pinyin, is the basic unit of standard Chinese phonetic structure. In general, a Chinese character represents a syllable. A syllable consists of three parts: the initial, the final, and the tone. “Initial” and “final” are terms used in ancient Chinese studies, and they only exist in syllables, where they are assigned according to their position. The component of a syllable before the vowel is termed the initial, which refers to the consonants at the beginning of the syllable. The final is the part of a syllable after the initial, consisting of “one to three vowels” or “vowels plus nasal consonants.” For example, “nian” is a syllable in standard Chinese, wherein “n” is the initial and “ian” is the final. Tone is the pitch change attached to the initial-final structure, which plays a role in discriminating semantics. There are four tones in standard Chinese: a high-level tone (Tone 1), a mid-rising tone (Tone 2), a low falling-rising tone (Tone 3), and a high-falling tone (Tone 4). For example, the same initial-final structure “nian” signifies different meanings in different tones: with tone 1 it means to pick up (拈ni**ā**n), with tone 2 it means year (年ni**á**n), with tone 3 it means to oust (撵ni**ǎ**n), and with tone 4 it means to miss (念ni**à**n).

Chinese characters are ideograms and there are no phonograms or grapheme-phoneme correspondence rules in Chinese (12–15). There are around 13,000 Chinese characters in the most widely used modern Chinese dictionaries; nevertheless, on average, roughly 15 characters share the same pronunciation, which are known as homophones (Standards Press of China, 1994). The majority of Chinese words are compound words and are made up of two characters (74%), which effectively eliminates the ambiguity caused by homophones (14). Chinese characters are square-shaped fonts that can be divided into single-component and compound characters, based on their structure. Chinese characters generated directly by the spatial arrangement of strokes are known as single-component characters, which evolved from pictures and signs. For example, the character “口” (kŏu/mouth) looks like a mouth in appearance. When a Chinese stroke “horizontal” (一) which indicates “speech” is added in the middle of “口,” it is written as the character “曰” (yūe) and means “to say.” After modification of the forms and structures, most single-component characters have been used as Chinese radicals to form compound characters. Two or more single-component characters can be combined according to their meaning to form an associative compound. For example, the combination of “不” (bù/not) and “正” (zhèng/straight) can form “歪” (wāi) to represent “crooked.” In addition, more than 80% of commonly used modern Chinese characters are composed of a semantic radical that provides clues to the general meaning category and a phonetic radical that indicates how the character is to be pronounced (15, 16). This is the so-called pictophonetic character. For example, using “木” (mù/wood) as a semantic radical can form characters related to trees such as “桃” (peach), “梅” (plum), “梨” (pear), and the phonetic radical “冈” (gāng/ridge) can form characters with the same pronunciation “gāng” such as “刚” (solid), “岗” (ridge), “钢” (steel). However, because of the historical evolution of phonology and semantics, ~13% of semantic radicals have lost their ideographic function, and only ~37.51% of pictophonetic characters have the same pronunciation as their phonetic radicals and are considered regular characters (17). Conversely, irregular characters have different tones, finals, or are wholly unrelated to their phonetic radicals. Radicals are divided into smaller and indivisible units for character font processing based on visual-spatial/motoric units, that is, components/logographemes (14). For example, the Chinese character “想” (xiǎng/think) consists of two radicals, “相” and “心,” which can be further broken down into three components “木,” “目,” and “心.” A Chinese character usually represents a syllable and a Chinese morpheme, forming the characteristic unity of shape, sound, and meaning that Chinese characters have.

## Methods

### Search strategy

We conducted a systematic review in accordance with the Preferred Reporting Items of Systematic Reviews and Meta-Analysis (PRISMA) guidelines. We systematically searched PubMed, Web of Science, and the Chinese medical databases Wan Fang Database and China National Knowledge Infrastructure (CNKI) to locate all case reports, case series, and treatises on PPA that have been published since 1994. Keywords used for retrieval included “primary progressive aphasia,” “progressive non-fluent aphasia,” “semantic dementia,” “logopenic aphasia” and specified terms like “Chinese,” “China,” and “Cantonese.” Two authors (JL and SO) independently assessed the definitions of PPA. All articles were read carefully and the reference lists were scanned for potential cases to include.

### Selection criteria

**Figure 1:**
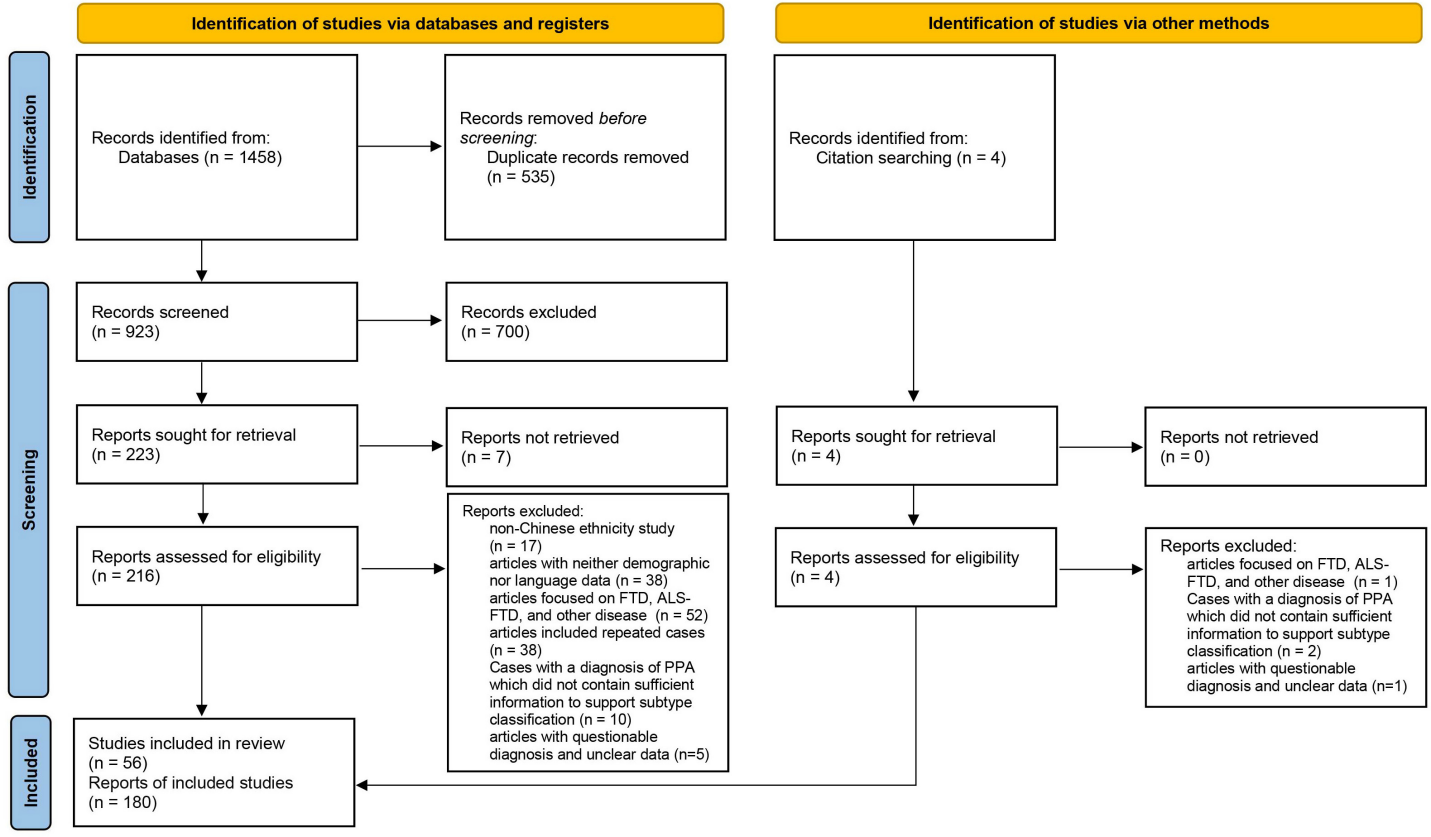
PRISMA flow diagram illustrating the selection of the studies.

The cases included in this review were required to fulfill the basic PPA criteria proposed by Mesulam (18, 19). We adopted the subtype classification of cases proposed by the original authors. Cases with a definitive diagnosis of PPA but no reported subtypes were reclassified according to the consensus criteria published in 2011 (2). The exclusion criteria were as follows: (1) the patients were not of Chinese ethnicity; (2) articles with neither demographic nor language data; (3) the study subjects were classified as FTD (no subtype classification), Amyotrophic lateral sclerosis (ALS), ALS-FTD (no subtype classification), and other diseases, or the variants of cases were behavioral variant frontotemporal dementia (bvFTD) and right temporal lobe variant of semantic dementia (RTLV); (4) cases with a diagnosis of PPA published between 1994 and 2011, which did not contain sufficient information to support subtype classification; and (5) articles with questionable diagnosis and unclear data. If the same cases were reported in several publications, they were counted only once. Overall, 180 cases from 56 publications were included in this systematic review (Figure 1) (20).

### Data collection and analysis

Demographic and clinical information collected included age at onset and recruitment, sex, disease duration, level of education, clinical manifestations of cognitive function, psychobehavioral symptoms, neurological signs (parkinsonism, supranuclear gaze palsy, motor neuron features), and language features (reading, writing, spontaneous speech, repetition, single-word and sentence comprehension, confrontation naming, and grammar), Mini-Mental State Examination (MMSE) scores, and neuroimaging. Cases that underwent examinations on three or more of the six language domains (reading, writing, repetition, comprehension, naming, and grammar) were included in the analysis of clinical manifestations. All analyses were performed using the R software (version 3.6.2). Continuous variables are described using mean (standard deviation, SD) or median [interquartile range, IQR] with analysis of variance (ANOVA) or Kruskal-Wallis H test to compare differences between groups. Categorical variables are described using the number of cases (percentage). Differences in distribution between the groups were compared using the corrected chi-square test or Fisher's exact test. Pairwise comparisons between groups for continuous variables were performed using the SNK-q test (normal distribution) or Benjamini and Hochberg (BH) adjusted Dunn's multiple comparisons (non-normal distribution), and pairwise comparisons between categorical variables were performed using the chi-squared partition. An FDR-adjusted *p*-value was used for *post-hoc* comparisons.

## Results

### Demographic features and cognitive assessments

Table 1 shows the demographic data and neuropsychological test results of the nfvPPA, svPPA, lvPPA groups. Sixteen patients (five with nfvPPA, four with svPPA, and seven with lvPPA) with detailed clinical manifestations lacked respective demographic data and thus were not included here. In addition, three cases were classified as unclassified PPA (21, 22). A total of 161 patients were therefore included in Table 1. The three groups were comparable in terms of sex, age of onset and recruitment, disease duration, educational level, and general cognitive assessment scores.

**Table 1:** Demographic characteristics and clinical scores in patients with PPA variants.

**Variables**	**ALL**	**nfvPPA**	**svPPA**	**lvPPA**	***p*** **-Value**
	***n*** **= 161**	***n*** **= 22**	***n*** **= 123**	***n*** **= 16**	
Sex					0.443
Male	93 (57.8%)	14 (63.6%)	72 (58.5%)	7 (43.8%)	
Female	68 (42.2%)	8 (36.4%)	51 (41.5%)	9 (56.2%)	
Age at onset (years)	61.5 (8.18)	61.6 (10.5)	61.9 (8.07)	58.4 (4.22)	0.272
Age at recruitment (years)	64.4 (8.43)	64.6 (10.4)	64.7 (8.39)^a^	61.8 (5.29)	0.411
Disease duration (years)	3.00 [2.00;4.00]	3.00 [2.00;4.00]	3.00 [2.00;4.00]^a^	2.00 [1.75;5.00]	0.962
Educational level					0.547
Illiteracy	5 (3.36%)	0 (0.00%)	5 (4.31%)	0 (0.00%)	
Primary school	18 (12.1%)	4 (23.5%)	13 (11.2%)	1 (6.25%)	
Secondary school	76 (51.0%)	7 (41.2%)	62 (53.4%)	7 (43.8%)	
College	50 (33.6%)	6 (35.3%)	36 (31.0%)	8 (50.0%)	
MMSE scores (range 0–30)	18.0 [11.0;23.0]^b^	20.5 [7.50;25.8]^c^	18.0 [11.5;23.0]^d^	13.0 [9.00;20.2]^e^	0.303

Data are represented as mean (SD), median [IQR], or n (%). nfvPPA, non-fluent variant primary progressive aphasia; svPPA, semantic variant primary progressive aphasia; lvPPA, logopenic variant primary progressive aphasia; MMSE, Mini-Mental State Examination.

a n = 120,

b n = 123,

c n = 18,

d n = 91,

e n = 14.

### Features of language and other cognitive impairments

Table 2 shows features of language impairments and other cognitive and behavioral domains in each PPA type. Thirty-three patients (4 nfvPPA, 29 svPPA) with detailed demographic data but no comprehensive clinical manifestations were not included here.

**Table 2:** Clinical manifestations of patients with PPA variants.

**Variables**	**nfvPPA**	**svPPA**	**lvPPA**	***P*** **-value**	**Adj.p^a^**	**Adj.p^b^**	**Adj.p^c^**
	***n*** **= 23**	***n*** **= 98**	***n*** **= 23**				
Pronunciation distortion	12/22 (54.5%)	1/56 (1.8%)	3/22 (13.6%)	**< 0.001**^a^, ^b^	< 0.001	< 0.05	0.118
Phonological paraphasia	9/22 (40.9%)	0/56 (0.0%)	14/22 (63.6%)	**< 0.001**^a^, ^c^	< 0.001	0.227	< 0.001
Word finding difficulties	8/22 (36.4%)	32/56 (57.1%)	13/22 (59.1%)	0.206			
Semantic paraphasia	3/22 (13.6%)	9/56 (16.1%)	3/22 (13.6%)	0.944			
Impaired auditory comprehension	17/23 (73.9%)	74/79 (93.7%)	18/23 (78.3%)	**0.016** ^a^	0.063	1.000	0.110
Naming errors	18/22 (81.8%)	98/98 (100.0%)	22/23 (95.7%)	**< 0.001** ^a^	< 0.001	0.428	0.428
Repetition impairments	20/21 (95.2%)	38/84 (45.2%)	21/21 (100.0%)	**< 0.001**^a^, ^c^	< 0.001	1.000	< 0.001
Dyslexia	11/16 (68.8%)	66/86 (76.7%)	12/20 (60.0%)	0.291			
Dysgraphia	15/20 (75.0%)	39/60 (65.0%)	7/13 (53.8%)	0.452			
Agrammatism in speech	14/14 (100.0%)	1/23 (4.4%)	7/10 (70.0%)	**< 0.001**^a^, ^c^	< 0.001	0.118	< 0.001
Impaired grammatical comprehension	17/19 (89.5%)	0/81 (0.0%)	0/10 (0.0%)	**< 0.001**^a^, ^b^	< 0.001	< 0.001	-
Episodic memory loss	10/17 (58.8%)	47/82 (57.3%)	13/15 (86.7%)	0.097			
BPSD	9/18 (50.0%)	61/78 (78.2%)	11/16 (68.8%)	0.052			
Neurological positive signs	5/10 (50.0%)	2/25 (8.00%)	1/12 (8.3%)	**0.008**	0.058	0.132	1.000

Data are represented as %. nfvPPA, non-fluent variant primary progressive aphasia; svPPA, semantic variant primary progressive aphasia; lvPPA, logopenic variant primary progressive aphasia; BPSD, behavioral and psychological symptoms of dementia.

Bold values denote statistical significance at the p < 0.05 level.

Significantly different:

a nfvPPA vs. svPPA;

b nfvPPA vs. lvPPA;

c svPPA vs. lvPPA.

For post-hoc comparisons, significance is set at p < 0.05.

All patients with nfvPPA had early onset non-fluent expression difficulty with reduced, slow, effortful, and halting speech. Some also exhibited sound errors, abnormal intonation, impaired volume control, and speech that lacked information. The nfvPPA group had more marked pronunciation distortions and impaired grammatical comprehension than the other two groups. Patients with nfvPPA had more prominent phonological paraphasia, repetition impairments, and agrammatism than patients with svPPA. The majority of patients with nfvPPA showed syntactic errors, such as short sentences, simple or impaired structures, lack of function words, and word order errors.

Patients with svPPA spoke fluently and exhibited anomia, impaired word comprehension, and word-finding difficulties. They lost semantic knowledge of nouns, verbs, color, and shape at an early stage, and some of them showed category-specific semantic deficits (23). Therefore, they lacked notional words and spoke with empty contents. It should be noted that the auditory comprehension impairments of svPPA were mainly for words, and those of nfvPPA and lvPPA were mainly for sentences. Patients with svPPA had more naming errors than patients with nfvPPA. Besides, one svPPA patient made word order errors in writing, such as writing “银行” (bank) as “行银.” A small percentage of patients' initial symptoms also included facial agnosia or memory decline.

Patients with lvPPA initially presented with word-finding difficulties, naming errors, and frequent phonological paraphasia, with about half of them having less fluent speech. Seven patients with lvPPA exhibited simply structured spontaneous speech. Patients with lvPPA had more prominent phonological paraphasia, repetition impairments, and agrammatism than patients with svPPA.

There were no significant differences in word-finding difficulties, semantic paraphasia, dyslexia, dysgraphia, episodic memory loss, and behavioral and psychological symptoms of dementia (BPSD) among the three groups, and no significant difference in impaired auditory comprehension and neurological positive signs in the multiple comparisons analysis.

### Neuroimaging features in each PPA type

The neuroimaging results are shown in the Supplementary Table 1. Twenty-seven patients (six with nfvPPA, fourteen with svPPA, and seven with lvPPA) without images were not included here. Approximately half of the nfvPPA patients presented with left frontal and temporal lobes atrophy/hypometabolism, which was much greater in the left inferior frontal gyrus (4, 24, 25). The other half showed bilateral asymmetric atrophy/hypometabolism of the frontal and temporal lobes, which was more pronounced on the left side. Lesions in the left temporal lobe, particularly the temporal pole, were more common in patients with svPPA, with 14 patients having more severe lesions on the right side. The most prominent area of involvement in patients with lvPPA was the left temporoparietal area.

### Features of reading impairments in Chinese PPA

The types of reading errors are listed in Table 3. The types of reading errors reported in Chinese-speaking patients with PPA included tonal errors, regularization errors, visually related errors, semantic errors, phonological errors, unrelated errors, and non-response. Because there were not many detailed reports, we could only provide the type of errors, not the frequency. The information provided by the case-control studies that were not included is shown in Table 3.

**Table 3:** Types of reading errors of patients with PPA variants.

**Error type**	**nfvPPA**	**svPPA**	**lvPPA**
Tonal errors	++	(++)	(+)
Regularization errors	(+)	++	(+)
Visually related errors	(+)	++	+
Semantic errors	?	+	+
Phonological errors	+	-	+
Unrelated errors	?	+	?
Phonological dyslexia	-	-	-
Non-response	+	+	?

+=present, ++=present commonly, -=not present, ?=unclear. Symbols in parentheses represent the information provided by non-included case-control studies. nfvPPA, non-fluent variant primary progressive aphasia; svPPA, semantic variant primary progressive aphasia; lvPPA, logopenic variant primary progressive aphasia.

The study of reading in svPPA was the most comprehensive among the three subtypes. Reading errors in Chinese patients with svPPA can be classified into five types:

(1) Regularization errors: Regularization errors are further classified into two subclasses. The first is called “legitimate alternative reading of components (LARC) errors,” which refers to misreading an irregular Chinese character into one of its pronounceable components that is inappropriate for the target character but is legitimate and more typical (16, 26, 27). For example, misreading “腔” (qiāng/cavity) as its phonetic radical “空” (kōng/empty), and misreading “笔” (bǐ/pen) as its semantic radical “毛” (máo/fur) (5, 11). On the other hand, there are more than 250 heteronyms that have more than one pronunciation with their respective meanings in 3,500 commonly used Chinese characters. The second type of regularization error refers to the situation in which patients read the target character as one of its other pronunciations, e.g., the character “的” was pronounced as “dī” when it was supposed to read as “dē” (28).
(2) Visually related errors: Visually related errors occur when the output corresponds to a character that is orthographically similar to the target (29). For instance, misreading “旱” (hàn/drought) as a visually similar character “早” (zǎo/morning) (7).
(3) Semantic errors: Semantic errors occur when the output and target characters are semantically related (15, 30), such as the confusion of “刷” (shuā/brush) and “扫” (sǎo/sweep) (10).
(4) Unrelated errors: For example, “盐” (yán/salt) read as “gui” (created by the patient).
(5) Non-response.

Among these error types, regularization errors were the most common in svPPA. Some patients were able to read characters aloud correctly without comprehending meaning (7), while others showed a better understanding of words/characters and instructions than ability to read them aloud (31). Another interesting phenomenon was that some patients directly judged the meaning of the Chinese characters as the meaning of their radicals.

Errors reported in patients with nfvPPA included tonal errors, phonological errors, and non-response. Tonal errors signify that the pronunciation of the output and target differed only in tone (9). For example, “年” (nián/year) read as “念” (niàn/miss). Compared with controls, nfvPPA patients performed very poorly in all tone production tasks, such as reading out loud a set of characters with similar initial-final structure but different tones. Phonological errors mean that the response and the target share at least half of the phonetic features (initial-final structures) (29). For example, “年” (nián/year) read as “娘” (niáng/mother). Tonal errors were unique to Chinese patients with PPA, and patients with nfvPPA tended to make more tonal errors than phonological errors (9). The reading comprehension of words/characters was better than that of sentences. Although some patients failed to read words/characters aloud, they could understand their meanings (4, 25).

Reading errors in patients with lvPPA included visually related errors, semantic errors, and phonological errors (8, 32).

### Features of writing impairments in Chinese PPA

Most PPA patients with dysgraphia showed better ability to write their own names and addresses, and copy characters in writing examination, but had difficulties in dictation and spontaneous writing. PPA may only affect sophisticated tasks such as dictation at first, but as the disease progresses, deterioration will become obvious in other tasks. Although the majority of papers have reported PPA patients' writing impairments, thorough reports on error types and probabilities are uncommon.

The types of writing errors are presented in Table 4. The types of writing errors reported in Chinese-speaking patients with PPA included phonologically plausible errors, orthographically similar errors, semantic errors, compound word errors, sequence errors, unrelated errors, non-character errors, and non-response. Overall, writing errors can be classified into two broad categories: non-character responses and incorrect character responses.

**Table 4:** Types of writing errors of patients with PPA variants.

**Error type**	**nfvPPA**	**svPPA**	**lvPPA**
Non-character errors			
Stroke errors	**+**	**+**	+
Radical/component errors	**+**	**+**	+
Visual errors	**+**	(+)	+
Pictograph errors	**-**	**+**	-
Dyskinetic errors	**+**	**-**	+
Spatial errors	**+**	**-**	-
Phonologically plausible errors	**+**	**+**	+
Orthographically similar errors	**+**	**+**	?
Semantic errors	**+**	**+**	+
Compound word errors	**+**	(+)	+
Sequence errors	**?**	**+**	?
Unrelated errors	**+**	**+**	+
Non-response	**+**	**+**	+

+=present, -=not present, ?=unclear. Symbols in parentheses and the results on lvPPA are based on the findings provided by non-included case-control studies. nfvPPA, non-fluent variant primary progressive aphasia; svPPA, semantic variant primary progressive aphasia; lvPPA, logopenic variant primary progressive aphasia.

Non-character responses mainly include the following 6 types:

(1) Stroke errors are easily identifiable when the target strokes are deleted, added, substituted, and transposed.
(2) Radical/component errors indicate that the target radicals/components have been deleted, added, substituted, and transposed.
(3) Visual errors manifest as errors in which the radicals/components are substituted by non-existent radicals, but the output visually resembles the target.
(4) Pictograph errors refer to the substitution of Chinese characters with pictogram symbols, e.g., a patient drew an umbrella when dictating the character “伞” (umbrella) (7).
(5) Dyskinetic errors are caused by dyskinesia in the writing hand, resulting in relatively intact glyphs with interrupted, incomplete, or disproportionate strokes.
(6) Spatial errors mean that the radical is placed in an inaccurate position, and the spatial position between the radicals is enlarged as if there are two independent Chinese characters.

Incorrect character responses are defined as real characters included in the Modern Chinese dictionary, but not the target character. These can be subdivided into the following six types:

(1) Phonologically plausible errors primarily refer to a writing phenomenon corresponding to surface dyslexia in Indo-European languages, also known as surface dysgraphia, which refers to dictating exception words following sound-to-spelling conversion rules (33). In Chinese, they mainly denote characters that are homophonic or phonologically similar to the target, including those that differ only in their tone. Most of the target characters were replaced by higher frequency characters, e.g., “架” (jià/shelf) was replaced by “价” (jià/price), and “访” (fǎng/visit) was replaced by “反” (fǎn/contrary) (7, 9).
(2) Orthographically similar errors may be caused by stroke or radical/component errors. For example, the character “月” (moon) was written as “目” (eye) in which a “horizontal” was added, and the compound character “想” (think) was written as “相” (phase), in which the radical “心” (heart) was deleted (7). In addition, characters with similar structures are also part of this range, e.g., “去” (go) and “生” (get) (4).
(3) Semantic errors refer to the output and the target having similar or relevant meanings, such as writing “岁” (age) as “年” (year) (4).
(4) Compound word errors describe errors in which the target is substituted by another character of a compound word. For example, the character “整” was written when patients were asked to write “齐” of the compound word “整齐” (neat) (9).
(5) Sequence errors refer to reversal of the sequence of stroke writing.
(6) Unrelated errors indicate that patients write characters that are not phonologically, orthographically, or semantically similar to the target characters.

Writing errors found in nfvPPA patients comprised phonologically plausible errors, orthographically similar errors, semantic errors, compound word errors, unrelated errors, non-character errors (stroke errors, radical/component errors, visual errors, dyskinetic errors, and spatial errors), and non-response. Patients with svPPA showed phonologically plausible errors, orthographically similar errors, semantic errors, sequence errors, unrelated errors, non-character errors (stroke errors, radical/component errors, pictograph errors), and non-response. Unfortunately, there were no detailed reports of patients with lvPPA amongst the cases studied here.

## Discussion

### Demographic features of Chinese-speaking PPA

Statistically, the prevalence of PPA is approximately three cases per 100,000 (34, 35), while the prevalence of PPA in China has not yet been reported. The number of patients with svPPA in our study was significantly larger than that of other subtypes. While there is no agreement concerning which subtype of PPA is the most common: a multicenter study from France suggested that the most common subtype is lvPPA (34), whereas nfvPPA predominates in our research in Japan (36). Since our study was more affected by publication bias, in that svPPA patients are more suitable for case reports due to their characteristic language impairments compared to other PPA subtypes, it is preferable to refer to other Chinese case-control or cohort studies. A study from Shanghai included three times more patients with svPPA than with nfvPPA (37). However, a study from North China enrolled the same number of nfvPPA patients as svPPA patients (38), and a Northeast Chinese Master's study included more patients with lvPPA. Thus, we cannot conclude that svPPA is the most common subtype in China. Studies have shown that the typical age of onset of PPA ranges from 50 to 70 years, with an average age of onset in the late fifties and nearly equal prevalence in both sexes (1, 39–41). The mean age of PPA onset in our study was slightly higher, with a slight male predominance.

### Features of language and other cognitive impairments in each PPA type

The core symptoms of Chinese patients with PPA were the same as those of Indo-European- and Japanese-speaking patients. Sound-level errors in nfvPPA were caused by both apraxia of speech and/or phonological paraphasia, which is consistent with previous research (42). Impairments in auditory comprehension and repetition in patients with nfvPPA were mainly caused by agrammatism. Moreover, impaired speech motor planning and the subvocal rehearsal component may contribute to repetition deficits. In svPPA, this may be due to the disintegration of semantic representations, and in lvPPA patients, it may have been due to impaired short-term memory and phonological storage (43, 44). Studies have shown varying prevalence and extent of memory deficits for PPA variants, with evidence of widespread episodic memory loss in lvPPA patients (45). In terms of behavior changes, patients with svPPA exhibit significantly more behavioral disturbances than other PPA subtypes, including disinhibition, eating habit changes, stereotyped behavior, and empathy loss (35, 46). In our study, the probability of episodic memory loss did not differ significantly across PPA subtypes, although it was higher in lvPPA patients. In addition, there was no significant difference in BPSD, even though the percentage of neuropsychiatric symptoms in svPPA was higher among the three groups. The fact that less than half of the patients underwent neuropsychological tests, such as the Neuropsychiatric Inventory (NPI) could have influenced the results. Studies in the Indo-European language have shown that agrammatism in patients with nfvPPA is characterized by impaired production of verb inflection and verb argument structure, omission of function words, and reduced grammatical complexity (47, 48). Meanwhile, word order errors due to agrammatism in patients with nfvPPA aid in differential diagnosis (49). In Chinese, there are no restricted morphological changes (such as singular and plural, tense and subject-verb agreement). Therefore, the agrammatic error types in nfvPPA manifest mainly in word order, function words, and complexity in Chinese. The grammar ability of patients with svPPA has always been considered to be preserved (2, 48). Studies in Indo-European languages have proposed that the reduced performance of svPPA patients in word ordering tasks could be due to word comprehension deficits (49). Accordingly, word order errors in the writing of an svPPA patient in our case were considered be due to impaired semantics. Mild grammatical problems, such as reduced grammatical complexity, were also found in patients with lvPPA, which is consistent with previous studies (47, 50). Each subtype of PPA could show agrammatism, because agrammatism involves various symptoms, such as missing verbs, reduced sentence complexity, and omitted functional words, and is associated with a large neural network involving the left posterior middle temporal gyrus, inferior parietal lobe, inferior frontal gyrus, and their connecting fiber bundles (51–54).

### Features of reading and writing impairments in Chinese PPA

#### The function model of reading and writing in Chinese

**Figure 2:**
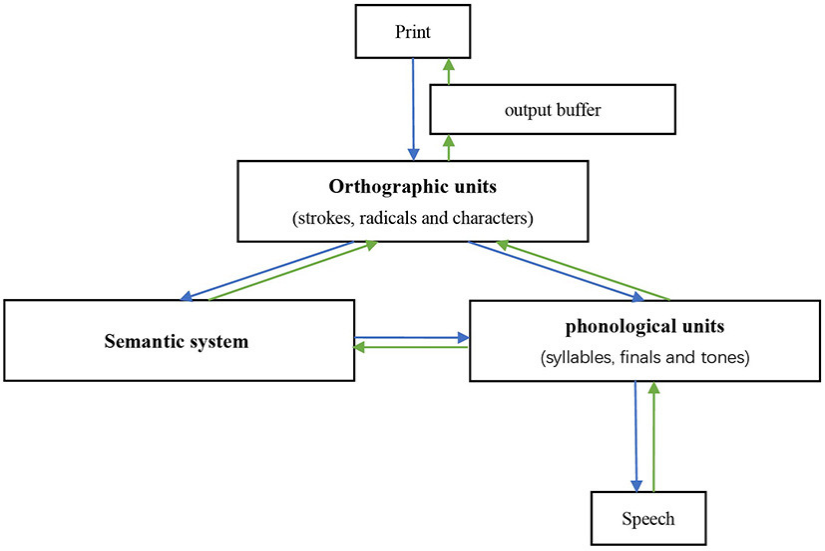
The “triangle model” of reading and writing in Chinese.

To better investigate the potential mechanisms, it is necessary to first introduce the functional models of reading and writing in Chinese, which differ from the dual-route model used in alphabet language (30, 55). The morphemic/syllabic level, rather than the phonemic level, is where Chinese characters map into language (29, 56). Unlike English words, which assemble phonemic units/syllables, the pronunciation of Chinese compound characters does not directly assemble the pronunciation of radicals and components. In other words, radicals/components do not correspond to the sub-syllabic units of phonological representations (57). Due to the above differences and the lack of grapheme-phoneme correspondence rules, a “triangle model” has been put forward, wherein reading in Chinese is proposed to depend on two independent and interrelated pathways (Figure 2): a lexical semantic pathway connecting orthographic units, semantic system, and phonological units, and a non-semantic pathway that contacts all orthographic representations (i.e., strokes, radicals, and characters) to all phonological representations (i.e., syllables, finals, and tones) bypassing the semantic system (13, 26, 30). Phonetic and semantic radicals have been proven to access their phonological and semantic representations in parallel with entire characters (16, 58). The neurocognitive process of writing to dictation is assumed to occur *via* reversal of these pathways (12, 15, 30). After the orthographic representations are retrieved, they are stored in an amodal “output buffer” until they are further processed (14).

#### The cognitive mechanism of tonal errors

The results showed that patients with nfvPPA presented with tonal errors in reading. Gorno-Tempini et al. observed that svPPA and lvPPA also exhibit tonal dyslexia (59). Patients with nfvPPA tend to make tone substitution errors, while patients with svPPA are prone to regularization errors in serial tone reading tasks (60). Furthermore, the accuracy of serial tone reading was much better in patients with lvPPA than in those with the other two subtypes. In addition, the performance of the tone-word matching test was poor in nfvPPA and svPPA patients, while that in lvPPA patients was relatively preserved (59). For example, patients made mistakes when they were asked to select the target that corresponded to the auditory stimulus from four Chinese characters with the same initial-final structure but different tones. Tonal errors are unique to Chinese patients and are not found in Japanese or English-speaking patients. Therefore, tonal tasks can be used as a potential diagnostic tool for Chinese-speaking PPA patients (9, 59, 60).

Pitch changes in tone are related to the anatomical properties of the speakers' vocal folds and larynx, and the fundamental frequency of vocal cord vibration per unit time. Due to the high prevalence of motor speech disorders, particularly apraxia of speech, in nfvPPA patients, it is not surprising that Chinese nfvPPA patients presented with tone dyslexia (61–63). For patients with svPPA and lvPPA, tonal errors were more likely to be caused by disorganized phonological codes. In terms of the triangle model, tonal errors are likely to result from a disruption of phonological units (12, 30). Law et al. proposed a phonology structure in which phonological representations have a multitiered form (12). Segmental features (i.e., consonants and vowels) and suprasegmental features (i.e., tones) are at the tiers of separation and generate connections independently of each other. Thus, once tonal information is disrupted, the suprasegmental tier will disassociate from the segmental tier, and a highly similar and intact representation with the same initial-final structure but different tone will be output or mapped into orthographic units instead of the impaired target.

#### The cognitive mechanism of errors on orthography

The results showed that both svPPA and lvPPA patients had visually related errors in reading. Patients with nfvPPA showed visual errors in writing, and orthographically similar errors, stroke errors, and radical/component errors were present in both nfvPPA and svPPA patients. In addition, the majority of stroke, radical/component, and visual errors retained the inherent configuration of Chinese characters. It is important to note that all three subtypes show these types of errors in reading and writing in practice. Whereas, visually related errors were more prominent in svPPA, radical/component errors were more common in nfvPPA, and visual errors and stroke errors were much more common in lvPPA (64, 65). In fact, visually related errors were also reported in Japanese patients with svPPA (66), and all three subtypes showed such writing errors in kanji character in our clinical work. Therefore, these types of errors are common in patients with PPA using logographic orthography.

The triangle model could account for visually related errors by implying that the mapping between print and orthographic units, or orthographic units *per se*, was faulty. Law et al. suggested that orthographic representations include not only the identities of the components and radicals, but also information on the structure of characters, perhaps in the form of structural templates or specification of position for each constituent (29). When the identity information of one of the constituents is selectively impaired, the system may fill in the missing information with another constituent based on intact structural information, such that the overall configuration of the character remains unaffected. Errors in writing involve additional speculations. Chinese characters are more dependent on orthographic working memory than English words due to their visuospatial intricacy (64). Therefore, disorders of the “output buffer” which contains graphic information (shape and/or stroke features) may result in these errors (14). Studies have shown that the dictation accuracy of Chinese patients with lvPPA decreases with increasing stroke numbers, and the left lingual gyrus is involved in this possess, which further supports this viewpoint (64).

#### The cognitive mechanism of regularization errors and phonologically plausible errors

The results showed that svPPA patients presented with regularization errors, and phonologically plausible errors were present in nfvPPA and svPPA patients. Studies have shown that regularization errors were found in all three subtypes of PPA, whereas they were more marked in svPPA (7, 65, 67). In addition, phonologically plausible errors were also present in lvPPA patients (64).

Similar to English- and Japanese-speaking svPPA patients characterized by surface dyslexia (2, 63, 66, 68–70), Chinese svPPA patients tend to make regularization errors in their reading. However, there was a little difference between all three. In English, patients assemble common pronunciations of phonemes or syllables directly to read irregular words, e.g., reading pint /paInt/ as /pInt/. In Japanese, about two-thirds of kanji characters have two or more different pronunciations, and the correct pronunciation depends on word/character collocation. Surface dyslexics assign a pronunciation that is wrong for the target word but legitimate for that character in other words (27). For example, reading “海老” (shrimp) /ebi/ as /kai/ and /rou/. In Chinese, regularization errors are divided into two subclasses. The second type manifests itself in the same way as surface dyslexia in Japanese; in both cases, the target character is read as one of its other pronunciations. The first (LARC errors) is different in that Chinese characters are read as one of its pronounceable radicals or components, that is, a constituent of the character font.

In terms of agraphia, patients with svPPA using Indo-European languages and Japanese tend to have phonologically plausible errors/surface dysgraphia (33, 68, 71, 72). Similarly, phonologically plausible errors are also frequently observed in Chinese svPPA (64). Phonologically plausible errors in Chinese can be classified into three types: errors that are homophonically or phonologically similar to the target, and errors that differ only in tone from the target. The first type is similar to surface dysgraphia in Japanese, in that both produce high-frequency and more common homophones at the lexical level. Instead of the phonologic regularity effect, writing accuracy in Chinese svPPA and lvPPA patients was associated with homophone density (64). Meanwhile, the performance of Indo-European-speaking patients exhibited a difference in that they dictated exception words following the sound-to-spelling conversion rules at the sublexical level, e.g., dictating “pint” as “paint.”

The triangle model suggests that regularization errors may be caused by selective lexical-semantic pathway impairment (13, 73). Radicals/components that emerge more frequently than entire characters might dominate phonological computation through the lexically mediated non-semantic pathway instead of the whole characters in the absence of adequate semantic constraints (15, 26, 30). Since Chinese is an opaque language with many homophones, the semantic system aids in eliminating ambiguity in orthographic output selection (74). However, phonologically plausible errors may occur when the impaired semantic system fail to provide appropriate semantic guidance (12, 30) and when relatively preserved phonological processing is overused (64). However, the essence of regularization errors and phonologically plausible errors in PPA patients who speak Chinese, Japanese, and English, is the loss of semantic knowledge; phenotypic differences only exist because of language differences.

#### Other reading errors

Compound word errors were observed in the patients with nfvPPA. Studies have shown that they can arise in all three subtypes, but more prominently in patients with nfvPPA. Due to the use of abundant compound words in Chinese and a correlation with the bilateral orbitofrontal gyrus, such errors may be secondary to the inability to inhibit the other characters of the two-character compound words (64). Furthermore, unlike English-speaking lvPPA patients that are characterized by phonological dyslexia, Chinese-speaking lvPPA patients were competent in reading pictophonetic pseudowords, such as the pseudoword “木冈” which consists of a semantic radical “木” and a phonetic radical “冈” (65). There are two possible reasons for this. First, pseudowords are made up of a phonetic radical and a semantic radical, and their pronunciation is consistent with that of the phonetic radical without the use of grapheme-phoneme correspondence rules. Therefore, patients can read this pseudoword depending on the phonetic radical. Second, Chinese characters are highly concentrated symbols with sound, form, and meaning, and are not susceptible to such errors. Finally, in addition to the error types mentioned in our results, Tee et al. also reported other rare errors such as phonetic radical errors, neographism, and perseveration dysgraphia (64).

## Limitation

Due to the inevitability of incomplete or missing data in a retrospective study, we can only provide the frequency of symptoms rather than the degree. The distribution of cases of each subtype was also affected by publication bias. In addition, we discovered that there are no unified language tests for PPA in China. In addition to the most commonly used Aphasia Battery of Chinese (ABC), researchers have adopted other scales such as the Western Aphasia Battery (WAB) and the Boston Diagnostic Aphasia Examination (BDAE). It is unavoidable to miss some of the less obvious symptoms owing to the lack of unified linguistic assessment tools and scoring criteria for patients with PPA. The absence of a standard impedes the comparability of patients from different studies for clinical and research purposes. Furthermore, there are no restricted morphological changes in Chinese, and word order and function words are the main ways to express grammatical relations. Therefore, it is more difficult to identify grammatical anomalies in Chinese than in Indo-European languages. Meanwhile, an anagram task (75), which is used in patients with severely reduced English language production, is absent from the Chinese grammatical assessment. Consequently, the description of agrammatism in articles is sometimes vague or lacking. Finally, none of the patients we gathered underwent pathological investigation, and fewer than 10 underwent lumbar puncture; therefore, the neuropathological changes that could result in PPA remain unknown. In conclusion, multicenter and multiregional research is expected to provide more comprehensive and detailed clinical data by employing a unified language task, which includes a detailed grammar examination.

## Conclusion

This paper provides the latest comprehensive demographic information on Chinese-speaking patients with PPA, summarizes their unique presentations in reading and writing, and investigates the underlying mechanisms for understanding PPA features in languages other than Indo-European languages. This review emphasizes the importance of establishing a standard diagnostic process across multicenter sites to form a large cohort, gain a more complete understanding of the full spectrum of PPA in Chinese patients, and improve diagnostic precision. More studies are expected to be conducted in Chinese-speaking patients with PPA to clarify the error frequency in reading and writing and their anatomical substrates.

## Data availability statement

The original contributions presented in the study are included in the article/Supplementary material, further inquiries can be directed to the corresponding author.

## Author contributions

KS and JL conceived the presented idea and framework for the systematic review. JL conducted the search for articles in consultation with KS and made the table and completed the PRISMA diagram. JL and SO analyzed the articles in consultation with KS. JL drafted the manuscript and KS and SO were involved in the planning of the manuscript. KS, SO, NK, and SK revised and provided feedback for the manuscript. All authors revised and approved the final manuscript.

## Funding

This work was supported by JST, the Establishment of University Fellowships toward the Creation of Science Technology Innovation, Grant Number JPMJFS2102. This work was also supported by Health Labour Sciences Research [Grant Nos. 20GB1002 and 20GC1008], Grant-in-Aid for Transformative Research Areas [Grant No. 20H05956], and Grant-in-Aid for Scientific Research (B) [Grant No. 21H03779] to KS.

## Conflict of interest

The authors declare that the research was conducted in the absence of any commercial or financial relationships that could be construed as a potential conflict of interest.

## Publisher's note

All claims expressed in this article are solely those of the authors and do not necessarily represent those of their affiliated organizations, or those of the publisher, the editors and the reviewers. Any product that may be evaluated in this article, or claim that may be made by its manufacturer, is not guaranteed or endorsed by the publisher.
