# Supplementary material for: Dyslexia and dysgraphia of primary progressive aphasia in Chinese: A systematic review
Source: Front Neurol. 2022 Dec 6;13:1025660. doi: 10.3389/fneur.2022.1025660 (PMC9764844; doi:10.3389/fneur.2022.1025660)
Supplement: Supplementary file 1 [file Table_1.docx]

**Supplement material Table 1. Imaging of patients with PPA variants.**

| **Regions of atrophy or hypometabolism** | **nfvPPA** | **svPPA** | **lvPPA** |
| --- | --- | --- | --- |
|  | **n=21** | **n=113** | **n=16** |
| CT | 1 | 2(XeCT*1) | 2(CTP*1) |
| MRI | 19 | 109 | 15 |
| SPECT | 2 | 10 | 2 |
| FDG-PET | 11 | 24 | 11 |
| Left frontal lobe | 18 | 27 | 11 |
| Right frontal lobe | 8 | 16 | 5 |
| Left temporal lobe | 16 | 108 | 13 |
| Right temporal lobe | 8 | 44 | 8 |
| Left parietal lobe | 5 | 7 | 9 |
| Right parietal lobe | 2 | 3 | 6 |
| Left occipital lobe | 0 | 1 | 5 |
| Right occipital lobe | 0 | 1 | 0 |
| Left temporoparietal junction | 0 | 0 | 9 |
| Right temporoparietal junction | 0 | 2 | 2 |
| Left hippocampus | 4 | 19 | 4 |
| Right hippocampus | 2 | 7 | 3 |
| Left insula | 3 | 6 | 0 |
| Right insula | 3 | 5 | 0 |
| Left caudate nucleus | 2 | 4 | 0 |
| Right caudate nucleus | 1 | 3 | 0 |
| Left anterior cingulate cortex | 2 | 0 | 0 |
| Right anterior cingulate cortex | 1 | 0 | 0 |
| Left basal ganglia | 1 | 0 | 0 |
| Left thalamus | 3 | 3 | 0 |
| Left occipitotemporal gyrus | 2 | 2 | 0 |
| Left parahippocampal gyrus | 0 | 1 | 0 |
| Right cerebellum | 1 | 0 | 0 |
| Enlargement of the left cerebral ventricles | 6 | 6 | 3 |
| Enlargement of the right cerebral ventricles | 1 | 2 | 1 |
| Widened left Sylvian fissures | 6 | 8 | 3 |

Note: Data are represented as n. nfvPPA: non-fluent variant primary progressive aphasia, svPPA: semantic variant primary progressive aphasia, lvPPA: logopenic variant primary progressive aphasia.
